# Stability of ACE2 Peptide Mimetics and Their Implications on the Application for SARS-CoV2 Detection

**DOI:** 10.3390/bios13040473

**Published:** 2023-04-13

**Authors:** Paula A. Santana, Claudio A. Álvarez, Santiago Valenzuela, Alberto Manchego, Fanny Guzmán, Cristian Tirapegui, Manuel Ahumada

**Affiliations:** 1 Instituto de Ciencias Químicas Aplicadas, Facultad de Ingeniería, Universidad Autónoma de Chile, el Llano Subercaseaux 2801, San Miguel, Santiago 8910272, Chile; 2 Laboratorio de Fisiología y Genética Marina, Centro de Estudios Avanzados en Zonas Áridas, Larrondo 1281, Coquimbo 1780000, Chile; 3 Facultad de Ciencias del Mar, Universidad Católica del Norte, Larrondo 1281, Coquimbo 1780000, Chile; 4 Laboratorio de Microbiología, Facultad de Medicina Veterinaria y Parasitología Veterinaria, Universidad Nacional Mayor de San Marcos, Lima 3673, Peru; 5 Núcleo Biotecnología Curauma, Pontificia Universidad Católica de Valparaíso, Valparaíso 2373223, Chile; 6 Escuela de Biotecnología, Facultad de Ciencias, Ingeniería y Tecnología, Universidad Mayor, Camino La Piramide 5750, Huechuraba, Santiago 8910272, Chile; 7 Centro de Nanotecnología Aplicada, Facultad de Ciencias, Ingeniería y Tecnología, Universidad Mayor, Camino La Piramide 5750, Huechuraba, Santiago 8910272, Chile

**Keywords:** ACE2 peptide, physical and chemical parameters, SARS-CoV-2 detection

## Abstract

The SARS-CoV-2 worldwide outbreak prompted the development of several tools to detect and treat the disease. Among the new detection proposals, the use of peptides mimetics has surged as an alternative to avoid the use of antibodies, of which there has been a shortage during the COVID-19 pandemic. However, the use of peptides in detection systems still presents some questions to be answered, mainly referring to their stability under different environmental conditions. In this work, we synthesized an ACE2 peptide mimic and evaluated its stability in different pH, salinity, polarity, and temperature conditions. Further, the same conditions were assessed when using the ability of the peptide mimic to detect the recombinant SARS-CoV-2 spike protein in a biotin-streptavidin-enzyme-linked assay. Finally, we also tested the capacity of the peptide to detect SARS-CoV-2 from patients’ samples. The results indicate that the peptide is structurally sensitive to the medium conditions, with relevance to the pH, where basic pH favored its performance when used as a SARS-CoV-2 detector. Further, the proposed peptide mimic was able to detect SARS-CoV-2 comparably to RT-qPCR results. Therefore, the present study promotes knowledge advancement, particularly in terms of stability considerations, in the application of peptide mimics as a replacement for antibodies in detection systems.

## 1. Introduction

The severe acute respiratory syndrome coronavirus-2 (SARS-CoV-2) has brought one of the worst pandemic events of recent decades, affecting 761,071,826 people and causing 6,879,677 deaths worldwide until March 2023 [1]. Besides its terrible effects on the population, the COVID-19 disease made humanity face the need to develop tools to detect it and fight back quickly. In this regard, vaccines represent the primary pathway to prevent the disease. Nevertheless, detecting the disease has proven to be the most arduous aspect of containment, as it necessitates an increased allocation of knowledge and resources [2]. Among the available techniques, the reverse transcription quantitative polymerase reaction (RT-qPCR) has been the gold standard for detecting SARS-CoV-2 in patients [3]. Nonetheless, other methodologies have been proposed to satisfy the high demand for detection kits. For example, mass spectrometry (MS), a technique that allows for separation, identification, and molecules quantification (based on their mass-to-charge relationship) [4], has been applied to SARS-CoV-2 identification by evaluating peptide fingerprints present in the virus without the need for its complete genome [5]. Another novel methodology has been the application of surface plasmon resonance (SPR), a technique that establishes specificity, affinity, and kinetic parameters during the interaction of different macromolecules and biomolecules with a metal surface through the measure of changes in the refractive index [6]. The classical enzyme-linked immunosorbent assay (ELISA) has also been used for SARS-CoV-2 detection due to its easy preparation, quick application, and low-cost equipment requirement [7]. In this sense, Freeman and collaborators [8] have already described, optimized, and validated a specific ELISA test for SARS-CoV-2 utilizing a prefusion-stabilized form of the spike protein [8]. Besides the proposition and demonstration of new detection systems, many fell short of solving one of the main problems: the shortage of antibodies/antigens necessary to form the immunocomplex with the virus.

In recent years, the synthesis and use of peptides has risen as a promising alternative to avoid using complex biomacromolecules. Synthetic peptides are smaller than proteins and can easily be synthesized in the laboratory and, later on, scaled up. Further, their sequence can be manipulated to specifically target biomolecules of interest, such as proteins in viruses, by precise and high-affinity interaction of their lateral chain’s functional groups [9]. Therefore, they have been used in developing new diagnosis systems, mainly for non-imaging diagnostics, such as ELISA, microarray, biosensors, and microfluidics [10]. Nonetheless, as with protein’s amino acid residues, peptide interactions are governed by many factors, such as surface charge and hydrophobicity distribution, pH, salt and buffer type, and ionic strength, among others, each influencing the peptide’s performance [11]. Thus, the peptide environment conditions in a solution can directly affect the diagnosis systems based on peptide target recognition. In this regard, although the use of synthetic peptides in diagnosis systems is expanding, some questions are still pending answers, such as how the test/sample environment could affect the peptide and, consequently, the diagnosis.

In this work, we have synthesized an ACE2 peptide mimic able to specifically bind the SARS-CoV-2’s spike protein based on the previous sequence reported by Zhang et al. [12] and then employed it in a biotin-streptavidin-enzyme-linked assay (bs-ELA) test using recombinant spike protein under different pH, polarity, salinity, and temperature conditions. Further, using patients’ nasopharyngeal samples with positive and negative SARS-CoV-2 RT-qPCR results allowed us to determine if ACE2 peptide mimic can recognize SARS-CoV-2 under different environmental conditions. The main objective was to evaluate the impact of the environment of the sample/test on the efficacy of ACE2 peptide mimic in detecting SARS-CoV-2, which could be translated to other systems using a synthetic peptide. At the same time, comprehending the factors leading to the peptide–SARS-CoV-2’s spike molecular interactions would contribute to a better understanding of the most predominant interactions under physiological conditions of SARS-CoV-2 with ACE2 cell receptors.

## 2. Materials and Methods

### 2.1. Materials

Sodium acetate, phosphate buffer saline (PBS) in tablets, 4-(2-Hydroxyethyl)piperazine-1-ethanesulfonic acid (HEPES), 2-Amino-2-(hydroxymethyl)-1,3-propanediol (Tris base), sodium hydroxide, Dimethyl sulfoxide (DMSO), Tween-20, and chloride acid were purchased from Sigma-Aldrich. Acetic acid, sodium carbonate, and sodium bicarbonate were obtained from Merck. All reagents were used as received, and dissolutions were prepared using Milli-Q water obtained from a water purification system Adrona CB1901.

### 2.2. ACE2 Analogous Peptide’s Synthesis and Characterization

ACE2 peptide mimic of 25 amino acid residues was prepared in plastic bags by the solid-phase peptide synthesis method, using the Fmoc/t-butyl strategy [12] on Rink amide resin (Iris) (0.65 mmol/g substitution). Cleavage and final deprotection were performed with a trifluoroacetic acid solution (TFA/H2O/triisopropylsilane/ethanedithiol) (92.5:2.5:2.5:2.5) (*v*/*v*/*v*/*v*) for 90 min at room temperature. Prior to coupling biotin to the peptide, ACE2 peptide mimic was cleaved from the resin to confirm the molecular mass of the peptide by electrospray ionization mass spectrometry (ESI-MS). Further, its purity was analyzed by reverse-phase HPLC with 0–70% acetonitrile–water mixture gradient for 8 min with a flow rate of 1 mL/min. After that, biotin was coupled using TBTU/HCTU/oxyme/DIEA activation. Then, the biotinylated peptide was precipitated with diethyl ether, extracted with water, and lyophilized. The obtained peptide was not subjected to any further purification and was used directly in subsequent trials.

### 2.3. Peptide Stability Evaluation

Peptide stability was evaluated considering different environmental conditions, including pH, osmolarity, polarity, and temperature. To this purpose, the following buffer solutions at 10 mM were used: acetate (pH 5.0), PBS (pH 7.0), HEPES (pH 7.0), Tris-HCl (pH 8.0), and bicarbonate (pH 8.0). Further, osmolarity influence was tested by using different NaCl concentrations (5, 10, and 25 mM) in the Milli-Q water. The dependence on the medium polarity was tested using DMSO at 10% *v*/*v* in PBS unless otherwise indicated. Finally, working temperatures chosen were 15, 25, and 37 °C.

The synthesized peptide’s circular dichroism (CD) spectroscopy was carried out on a JASCO J-815 CD Spectrometer coupled to a Peltier JASCO CDF-426S/15 system for temperature control (Jasco Corp., Tokyo, Japan). Spectra were recorded in the far ultra-violet (UV) range (190–250 nm) using quartz cuvettes of 0.1 cm path length and 1 nm bandwidth at 0.1 nm resolution. Each spectrum corresponds to an average of three repeated scans in a continuous scanning mode with 50 nm/min scanning speed with a response time of 1 sec. The contribution blank was subtracted from each spectrum. Molar ellipticity was calculated using 0.0007 mol/L of the peptide. CD spectra of the peptides were recorded in environmental conditions detailed above at 15, 25, and 37 °C.

Peptide’s tyrosine fluorescence was recorded in a Jasco FP-8300 spectrofluorometer (Jasco Corp., Tokyo, Japan). Excitation and emission wavelengths were 280 nm, and 290–400 nm, respectively. Both slit_ex_ and slit_em_ were used at 5 nm. Peptide samples were measured utilizing a quartz cuvette and considering the previously mentioned environmental conditions. Further, under the same experimental conditions, Ζ-potential values were measured by laser Doppler anemometry using a Zetasizer Advance Pro equipment (Malvern Instruments, Malvern, UK).

The biotinylated ACE2 peptide mimic was placed onto a 96-well MaxiSorp™ plate (Nunc), performing serial dilutions from 3.08 to 0.05 nM. The plate activation was performed considering different environmental conditions, including pH, osmolarity, polarity, and temperature (20 and 37 °C), as indicated in point 2.3 by overnight. Then, the plate was washed three times with PBS-Tween (PBS-T) 0.05%. Afterward, the wells were blocked with 3% bovine serum albumin (BSA) for 60 min at the corresponding temperature. Finally, the plate was washed thrice and incubated for 60 min with streptavidin-HRP (Thermofisher) from a stock (2 mg/mL) diluted at 1:10,000 (200 ng/mL) in PBS 1X. 100 μL per well of 3,3′,5,5′-tetramethylbenzidine (TMB) single solution (Invitrogen) was added and incubated for 30 min at room temperature; the absorbance was read at 650 nm with a VERSA max microplate reader (Molecular Devices, LLC, San Jose, CA, USA). All assays were performed in sextuplicate.

### 2.4. ACE2 Peptide as Sensing Probe for Recombinant SARS-CoV-2 Coronavirus Spike Protein under Different Conditions

The ability of biotinylated ACE2 peptide mimic to interact with the recombinant SARS-CoV-2 (2019-nCoV) Spike RBD protein (rSpike) (Sino Biological US Inc., Chesterbrook, PA, USA.) was evaluated by bs-ELA under different environmental conditions.

The rSpike was placed onto a 96-well MaxiSorp™ plate (Nunc), seeding 1, 0.75, and 0.5 µg per well (0.42 µM, 0.38 µM, and 0.21, respectively). The plate activation was performed with 10 mM PBS pH 7.2 or 50 mM carbonate–bicarbonate buffer pH 9.4 by overnight incubation at 4 °C. Then, the plate was washed thrice with PBS or sodium bicarbonate. Afterward, the wells were blocked with 3% BSA for 60 min at room temperature. Next, the plate was incubated with biotinylated ACE2 peptide mimic suspended in PBS or PBS containing 10% DMSO or sodium bicarbonate at 1 ng/mL of peptide concentration for 60 min. Finally, the plate was washed thrice and incubated with streptavidin-HRP from a stock (2 mg/mL) diluted at 1:10,000 (200 ng/mL) in PBS. 100 μL per well of TMB single solution was added and incubated for 30 min at room temperature; the reaction was read at 650 nm with a VERSA max microplate reader (Molecular Devices, LLC, San Jose, CA, USA). All assays were performed in sextuplicate.

### 2.5. ACE2 Peptide as Sensing Probe for Nasopharyngeal Samples of Patients with Positive and Negative SARS-CoV-2 Molecular Detection

The nasopharyngeal swabs (NpS) were obtained from the FIGEMA public university laboratory of SEREMI de Salud of Coquimbo, Chile (Res. 208, 17 April 2020). Samples were obtained from September to November 2020.

Total RNA was extracted from 200 μL of NpS using EZNA total RNA kit following the manufacture’s protocol. The SARS-CoV-2 RNA was detected in the NpSwab with a one-step RT-PCR method (BGI’s Real-Time Fluorescent RT-PCR kit for detecting 2019-nCoV(SARS-CoV-2), BGI Genomics), on Mx3000 Real-Time PCR System (Agilent, Santa Clara, CA, USA) following the manufacture’s protocol.

The ability of biotinylated ACE2 peptide mimic to recognize positive SARS-CoV-2 NpS was evaluated by bs-ELA as previously reported. For this, 50 µL of NpS samples were mixed with 100 µL of PBS 1X and then placed onto a 96-well MaxiSorp™ plate by overnight incubation at 4 °C. Then, the plate was washed thrice with PBS. The wells were blocked at room temperature with 3% BSA for 60 min. Next, the plate was incubated with ACE2 peptide mimic biotin-conjugated suspended in PBS at 1 ng/mL of peptide concentration for 60 min. Finally, the plate was washed thrice and incubated with 200 ng/mL streptavidin-HRP in PBS. Finally, 100 μL per well of TMB was added and incubated for 30 min at room temperature; the reaction was stopped with 50 μL of 1 N sulfuric acid and absorbance was read at 650 nm with a VERSA max microplate reader. All assays were performed in sextuplicate.

### 2.6. Statistical Analysis

Data were analyzed for statistical significance using the R version 3.5.2 software. Prior to statistical analysis, all data were tested for normality and homoscedasticity by Shapiro–wilk and Fligner–Killeen test, respectively. Two-way ANOVA followed by the Tukey post hoc test were used to compare environmental conditions on synthetic ACE2 peptide mimic stability, with buffer concentration and temperature as the main factors. In addition, one-way ANOVA and post-hoc Tukey’s multiple comparison tests were used to compare the effect of different saline buffer conditions on rSpike–ACE2 peptide mimic interaction. The results were expressed as mean value ± standard error of the mean (SEM) values measured in independent analysis. Differences were considered significant at *p* < 0.05 (*), *p* < 0.01 (**), and *p* < 0.01 (***). Data were graphically represented using GraphPad prism 8.1.

## 3. Results

### 3.1. ACE2 Peptide Mimic Characterization

**Figure 1:**
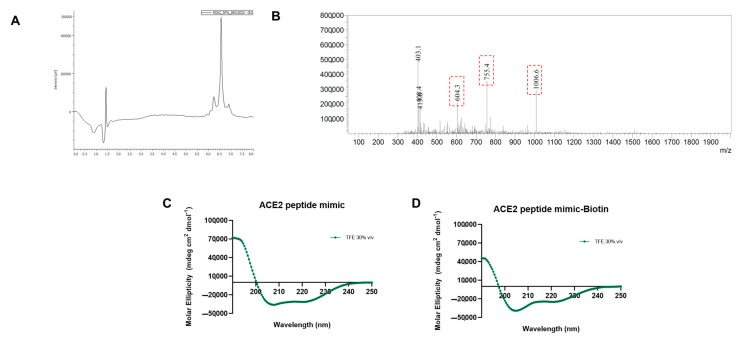
Mass and structure analysis of ACE2 peptide mimic. (**A**) RP-HPLC chromatogram of ACE2 peptide mimic, which was eluted at 6.7 min on a C18 column. (**B**) ESI-MS spectrum, showing the charged ion [M + 3H] ^3+^ (m/z 1006.4), [M + 4H] ^4+^ (m/z 754.8), and [M + 5H] ^5+^ (m/z 603.8) of synthetic ACE2 peptide mimic (MW 3019.204 Da). (**C**,**D**) Representative circular dichroism spectra for ACE2 peptide mimic and ACE2 peptide mimic biotin in trifluoroethanol 30% *v*/*v* in water.

The peptide was synthesized using the Fmoc strategy, and its purity and molecular mass were confirmed by RP-HPLC (Figure 1A) and ESI-MS (3019.204 Da; Figure 1B). Circular dichroism confirmed that ACE2 peptide mimic and ACE2 peptide mimic biotin tend to adopt α-helical structures with a double minimum between 205–225 nm and a maximum of around 190 nm (Figure 1C,D).

### 3.2. ACE2 Peptide Mimic Stability

**Figure 2:**
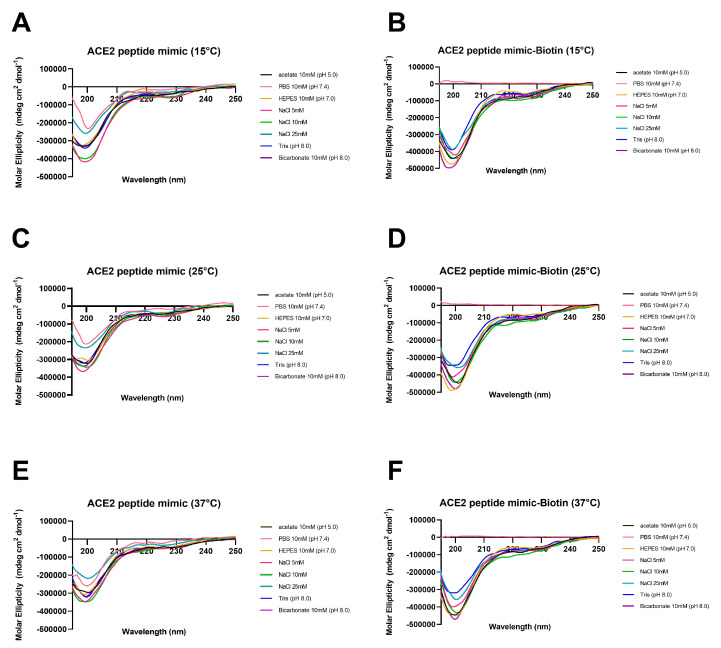
Effect of different environmental conditions on ACE2 peptide mimic stability. Circular dichroism spectra of synthetic biotinylated ACE2 peptide mimic and the non-biotinylated one were obtained at different temperatures—(**A**,**B**) 15 °C, (**C**,**D**) 25 °C, and (**E**,**F**) 37 °C—and different buffer solutions at 10 mM of acetate (pH 5.0), PBS (pH 7.0), HEPES (pH 7.0), Tris-HCl (pH 8.0), and bicarbonate (pH 8.0). Osmolarity influence was tested using different NaCl concentrations (5, 10, and 25 mM) in the Milli-Q water.

To compare the secondary structure of biotinylated ACE2 peptide mimic and the non-biotinylated one in different environmental conditions of pH, osmolarity, and polarity, as well as evaluate thermal stability, circular dichroism spectra were obtained. The results showed that biotinylated and non-biotinylated peptides have a random coil tendency since a minimum was observed at around 200 nm and a very weak negative band around 220–230 nm in all buffer solutions was evaluated at 15, 25, and 37 °C (Figure 2).

Tyrosine (Tyr) fluorescence was also assessed; this amino acid, present in our ACE2 peptide mimic sequence, has been described as a tool to evaluate protein conformational changes [13]. In this regard, the changes in the Tyr emission spectra, mainly when no tryptophan residue is present [14], allow the establishment of the peptide’s stability when stressed under different environmental conditions (Figure S1). Table 1 shows that in every tested condition, the increase in temperature decreased the Tyr fluorescence by approximately 20–30% when it rose from 15 °C to 37 °C, with the exemption of acetate, which decreased by around 40%. Further, the highest fluorescence was researched in DMSO, followed by HEPES and PBS buffers (pH 7), Tris and bicarbonate buffers (pH 8), and acetate buffer (pH 5). Moreover, significant differences were observed when the ionic strength of the medium increased as the fluorescence emission decreased by around 5–10% (PBS 10×).

Additionally, Table 1 shows the ζ potential values of the ACE2 peptide mimic under the different tested conditions. The higher and lower tested pH buffers showed the most negative values. On the other hand, measurements using buffers at pH 7 promoted values close to −8 mV. Finally, when using DMSO, it shows the closest to zero value.

**Figure 3:**
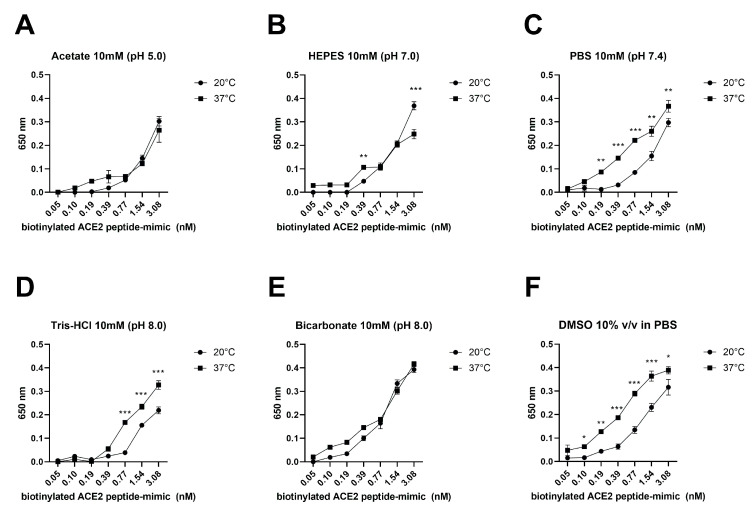
Synthetic ACE2 peptide mimic stability evaluation in different environmental conditions. The biotinylated ACE2 peptide mimic was placed onto a 96-well MaxiSorp™ plate, performing serial dilutions from 3.08 to 0.05 nM in (**A**) Acetate 10 mM pH 5.0; (**B**) HEPES 10 mM pH 7.0; (**C**) PBS 10 mM pH 7.4; (**D**) Tris-HCl 10 mM pH 8.0; (**E**) Bicarbonate 10 mM pH 8.0; (**F**) DMSO 10% *v*/*v* in PBS 1X at 20 and 37 °C. Data are given as mean ± SEM, and differences were considered significant when * *p* < 0.05, ** *p* < 0.01, *** *p* < 0.001 (Two-way ANOVA).

The behavior of biotinylated ACE2 peptide mimic in different solutions at 20 and 37 °C was studied using the enzyme-linked assay. Figure 3 shows that at 20 °C, the biotinylated ACE2 peptide mimic in a 10 mM acetate solution pH 5.0 could detect it from 0.39 nM, observing a direct proportionality between peptide concentration and absorbance (Figure 3A). Similar behavior was observed with HEPES 10 mM pH 7.0, PBS 10 mM pH 7.4, and Tris-HCl 10 mM pH 8.0 (Figure 3B–D). However, upon dissolving the peptide in 10 mM bicarbonate pH 8.0 or 10% *v*/*v* DMSO in PBS, peptide detection was achieved starting at 0.19 nM, where a direct proportionality between peptide concentration and absorbance was then observed (Figure 3E,F). Additionally, it was observed that the maximum absorbance reached, in ascending order and at the maximum peptide concentration (3.08 nM), was when the peptide was dissolved in Tris-HCl (0.22), PBS (0.29), acetate (0.3), DMSO (0.32), HEPES (0.37), and bicarbonate (0.39). On the other hand, at 37 °C, the detection of the biotinylated ACE2 peptide mimic in acetate and HEPES solutions was variable, and there was no direct proportionality between peptide concentration and absorbance (Figure 3A,B). On the contrary, when using PBS, a direct relationship was observed between absorbance and concentration starting at 0.05 nM (Figure 3C). In the case of Tris-HCl, bicarbonate, and DMSO, similar behavior was observed at both temperatures, with a slight absorbance increase when the assay was performed at 37 °C (Figure 3D–F). In none of the cases did the absorbance increase above 0.4 a.u. in absorbance at the maximum concentration of biotinylated ACE2 peptide mimic evaluated (3.08 nM).

### 3.3. Effects of Saline Composition and pH on Recognizing SARS-CoV-2 Coronavirus Spike Protein by ACE2 Peptide Mimic

**Figure 4:**
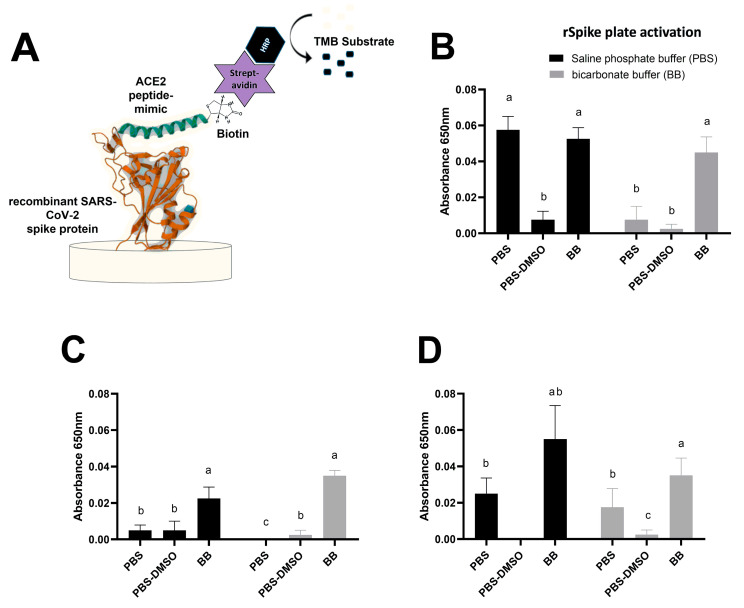
Measurement of rSpike-ACE2 peptide mimic interaction under different saline buffer conditions. (**A**) Schematic diagram of the bs-ELA experiment steps. (**B**–**D**) shows differences in the microplate activation with rSpike in PBS (black bar) or BB (grey bar) at 0.42 µM, 0.38 µM, and 0.21 µM, respectively. Differences were considered significant when *p* < 0.05 and indicated with a different letter after one-way ANOVA.

The two most widely used buffers in ELISA assays (PBS and BB) were used to evaluate the effect of salt composition and pH on the ability of the ACE2 peptide mimic to recognize the SARS-CoV-2 spike protein. Thus, the microplate was activated with this recombinant protein (Figure 4A) suspended in BB or PBS. Further, DMSO influence was also tested by incorporating it into the osmolyte. Finally, the reactivity was measured by forming the biotin–streptavidin complex, the latter conjugated to peroxidase (Figure 4A). The results showed a more significant interaction of the ACE2 peptide mimic spike protein when the amount of protein seeded in the plate was at least 0.42 μM (Figure 4B). Furthermore, this interaction was favored when PBS was used in the activation process, regardless of the buffer utilized later to suspend the ACE2 peptide mimic. However, when BB was used for the activation, keeping the same buffer for posterior steps with the peptide was necessary. Moreover, an apparent negative effect was also observed when adding DMSO in these buffers since its incorporation significantly decreased the spike protein detection by the ACE2 peptide mimic, independent of the buffer used.

### 3.4. Recognizing of SARS-CoV-2 Nasopharyngeal Swabs by ACE2 Peptide Mimic

**Figure 5:**
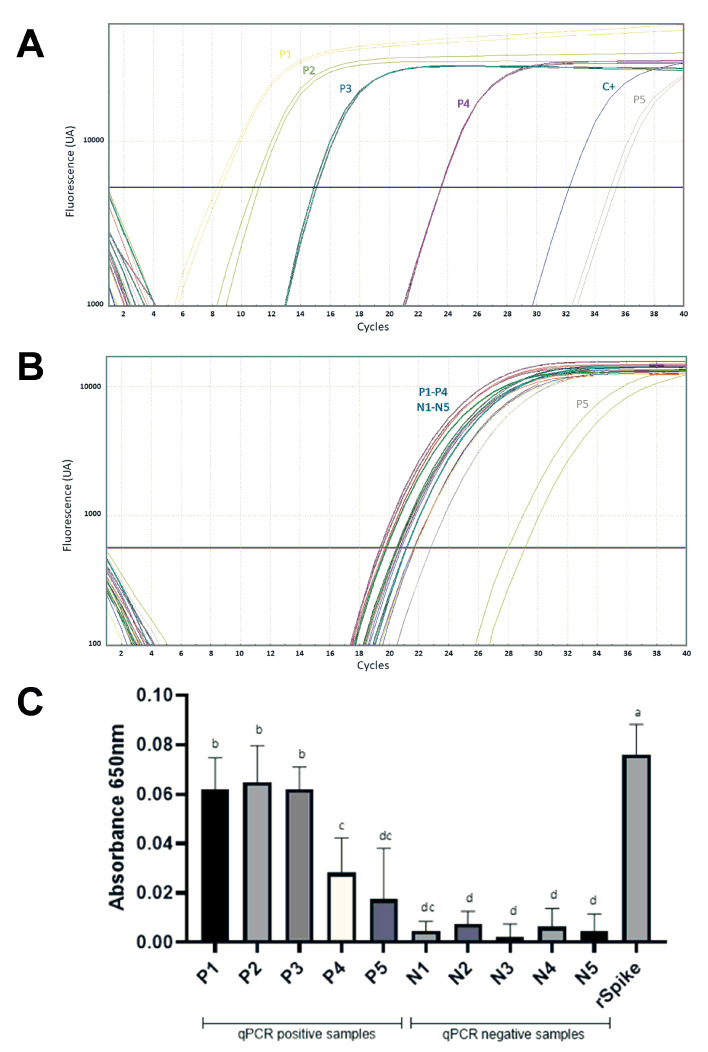
Comparative detection of SARS-CoV-2 on nasopharyngeal swabs by RT-qPCR and bs-ELA. (**A**,**B**) show the amplification curves of the ORF1ab gene of SARS-CoV-2 and β-actin gene in the nasopharyngeal swabs, respectively. Samples with positive detection of the genetic material of SARS-CoV-2 were named P1 to P5, whereas the negative RT-qPCR samples were named N1 to N5. (**C**) Detection of SARS-CoV-2 on nasopharyngeal swabs by biotin-streptavidin-enzyme-linked assay using ACE2 peptide mimic. Differences were considered significant when *p* < 0.05 and marked with a different letter after one-way ANOVA.

To analyze whether the ACE2 peptide mimic can recognize positive SARS-CoV2 nasopharyngeal swabs, samples containing different amounts of the virus were used, which were determined by analyzing the amplification cycles of the ORF1ab gene of SARS-CoV-2 (Table 1). The results showed that, under the evaluated conditions, there was a positive correlation between the detection of positive SARS-CoV-2 RTqPCR swabs and their detection by reactivity in bs-ELA using the biotinylated ACE2 peptide mimic (Figure 5A–C). However, for one of the positive samples containing the least amount of genetic material, its detection by bs-ELA failed to differentiate from the SARS-CoV-2 negative samples (Figure 5C and Table 2).

## 4. Discussion

New strategies for detecting virus macromolecules have been introduced to reduce the period of pathogen detection in biological samples and fomites on surfaces. Peptide-based sensors as synthetic biomimetics emerge as an alternative to the use of antibodies in protein detection systems [15], competing with strategies based on the detection of genetic material.

This work evaluated the ACE2 peptide mimic under different environmental conditions of pH, salinity, polarity, and temperature. In the first instance, an extensive evaluation related to its structure was performed. CD results indicated that in all the tested buffers (Figure 2), there was little to no presence of a secondary structure, as a general random coil trend could be observed [16]. However, in an apolar medium (30% TFE), the peptide adopted a clear helicoidal signal that can be assigned to an α-helix structure (Figure 1). Such a finding is not strange, as peptide mimics usually require hydrophobic environments to acquire the expected conformation. In this line, Zhang et al. [12] previously designed an ACE2 peptide mimic, finding that their peptide acquired an α-helix structure when in contact with the Spike protein. Related to the effect of the environmental conditions on the peptide’s CD spectra, the temperature increase promoted a decrease in the negative molar ellipticity, which has been previously attributed to changes in the secondary structure [17]. Similar results were observed for the salinity increase [18]. More remarkable findings were observed when comparing the pH, where its increase also promoted a gain in the negative signal of molar ellipticity with a little tendency to form an α-helix structure, which could be associated with the increase in the electrostatic repulsion derived from the carboxyl groups [19]. Further, there were observable differences for buffers at the same working pH. For instance, at pH 7, only the HEPES buffer generated a measurable signal that could be related to its zwitterionic behavior. It should be noted that using PBS solution during the CD measuring did not cause a measurable signal, particularly in the case of the biotinylated ACE2 peptide mimic. This has been previously reported and associated with a high-tension voltage value generated by this buffer [20]. Moreover, at pH 8, there was a significant difference in the CD spectra between the Tris-HCl and bicarbonate buffers, with this last one having a more substantial signal that could correlate to the tendency to secondary structure formation. We hypothesize that the pH plus the charge stabilization promoted by the bicarbonate salts give the best environmental condition to stabilize the structure of the ACE2 peptide mimic.

To further explore the effect of the environment on the molecular conformation of the ACE2 peptide mimic and its stability, we evaluated the tyrosine amino acid fluorescence present in the peptide sequence. Although there are differences in the measured fluorescence, it is known that tyrosine fluorescence is almost insensitive to the medium polarity [21], which explains why no shift in the wavelength at the maximum emission was observed in the different environments (Figure S1). Yet, its quenching can be explained by either ionization of the amino acids with concomitant amino or carboxyl groups or by energy transfer in the excited state [22]. Therefore, Tyr fluorescence has been actively proposed as a marker for peptide/protein conformational changes [13]. Thus, when comparing the temperature effect in all the tested mediums, the Tyr fluorescence decreased significantly, which can be attributed to a change in the structure conformation that brings it closer to other residues able to quench it. Despite the influence of temperature, the other conditions did not promote significant changes in the fluorescence, with two apparent exceptions. The first one relates to the exacerbated quenching in the acetate buffer, which has been previously associated with a proton transfer quenching mechanism [23], indicating that the mentioned buffer promotes structure instability. The second one is about the bicarbonate buffer where, besides its lower fluorescence (compared to other mediums), it is the buffer with the most downward change in emission across the tested conditions. Such an effect could be justified with respect to the alkaline pH and the peptide’s negative surface charge, which plays a crucial role in avoiding the ionization quenching mechanism and in stabilizing the molecule [24].

Regarding the ζ potential, i.e., peptide charge, in the present study, these are reported as average zeta potential values, as the high ionic strength of some of the buffers did not allow us to obtain the ζ distribution; similar phenomena have been reported elsewhere [25]. The theoretical net charge of the here synthesized peptide is 4.9 at pH 7, which is closely related to the obtained ζ value in 10% DMSO (see Table 1) and explained in terms of the favorable structural conformation induced by the polarity of the organic solvent [26]. Similarly, the peptide in buffers at pH 7 showed values higher than −10 mV; slight differences among them can be attributed to the presence of ions at the electrical double layer of the peptide. Conversely, pH values of 5 and 8 elicited more negative ζ values (<−15 mV). While pH is a determinator factor in ζ potential measurement [27], the chosen aqueous environment is also relevant. Notably, in the case of the bicarbonate buffer, the anionic charge is slightly moderated because of the presence of the counter ions (Na^+^). This is not the case for Tris-HCl and acetate buffers, which are composed mainly of a weak base and acid, respectively. Therefore, a divergence from the other systems is expected. However, such a difference, particularly for bicarbonate, also established a higher stability of the ACE2 peptide mimic in this aqueous medium, which aligns with the previously described results.

In terms of detecting the biotinylated ACE2 peptide mimic through the enzyme-linked assay in a microplate, it was determined that the use of PBS at 37 °C or bicarbonate at both temperatures improved the sensitivity of the assay, being able to detect the peptide from 0.05 to 3.08 nM. Similar behavior was obtained with 10% DMSO at 37 °C; however, when using this osmolyte, the curve saturated at 1.54 nM. Likewise, using human ACE2 receptor-derived mimetic peptides can improve the virus variant detection systems, considering that the most worrisome epidemiological mutations favor the spike protein’s binding with the mentioned receptor [28,29]. Further, our ACE2 mimetic-peptide could discriminate between patients’ positive and negative samples, yet the proposed detection system required samples with a high concentration of SARS-CoV-2 genetic material. In fact, such a finding was also observed when employing the recombinant spike protein, where the best detection signal was observed at 0.42 µM of rSpike. In this regard, other studies have shown higher sensitivities; for example, a recent work using an ACE2 mimetic peptide demonstrated the feasibility of developing highly sensitive methods such as a FRET-based detection system [30]. Additionally, Liu et al. (2021) [31] developed an electrochemical detection system for SARS-CoV-2 by functionalizing the electrode surface with a synthetic peptide to bind the host cell surface ACE2 receptor specifically. They showed that the proposed method could detect concentrations as low as 1 pM [31]. Nonetheless, it must be considered that under the presented conditions, the experimental needs are minimal compared to other studies. Furthermore, it is necessary to consider what conditions would favor virus recognition. Our work established that the best conditions for these interactions occurred at basic pH, using phosphate or bicarbonate buffer. In addition, it was observed that the presence of some osmolytes, such as DMSO, often used to improve the solubility of peptides and proteins and prevent their aggregation [32], can interfere in the interaction of ACE2 mimetic peptides with the spike protein, which would affect diagnostic systems based on this strategy. Thus, using other osmolytes in systems based on mimetic peptides should be considered to avoid new interferences.

## 5. Conclusions

The use of synthetic peptides in virus detection systems has emerged as a potential alternative to replace the use of antibodies. In this work, we have explored the stability of an ACE2 mimetic peptide to be employed in an enzyme-linked assay under different pH, polarity, salinity, and temperature conditions. The obtained results of those stability factors obtained by means of structural, conformational, and surface charge allow us to conclude that basic pH improves the peptide stability, particularly when using bicarbonate buffer because it provides charge stabilization, avoiding undesired side mechanisms such as ionization or proton transfer that ultimately destabilize the peptide. Further, the peptide stabilization under such conditions enabled us to detect the rSpike protein at a concentration of 0.21 μM, employing a peptide concentration as low as 0.05 nM in an enzyme-linked assay. Moreover, using the ACE2 mimetic peptide allowed us to discriminate between positive (with a high concentration of SARS-CoV-2 genetic material) and negative results in patients’ samples. While the here-explored detection limit is not as sensitive as other reported methods, it is a step forward in developing new detection systems for in situ applications, highlighting the relevance of peptide stability for such a development. Therefore, in our future work, we will explore ways to improve the sensitivity of peptide-based sensors by synthetic biomimetics that can be applied in situ.

## Figures and Tables

**Table 1:** Percentage of Tyr fluorescence emission and ζ potential values of ACE2 peptide mimic when exposed to different environmental conditions.

Solvent/Condition	% Tyr Fluorescence Emission *			ζ Potential (mV) **
	15 °C	25 °C	37 °C	
DMSO 10%	100 ± 1.2	89.8 ± 0.7	82.4 ± 4.7	−4.434
Acetate (pH 5)	85.8 ± 6.6	60.7 ± 4.1	53.5 ± 3.8	−23.6
PBS 1× (pH 7)	93.5 ± 5.4	75.6 ± 6.6	69.1 ± 6.3	−7.71
PBS 10× (pH 7)	87.0 ± 5.5	68.2 ± 2.8	59.8 ± 1.2	−9.155
HEPES (pH 7)	93.9 ± 3.9	75.5 ± 4.7	69.5 ± 2.2	−7.6
Bicarbonate (pH 8)	83.5 ± 7.1	73.3 ± 1.8	67.8 ± 3.1	−14.88
Tris-HCl (pH 8)	87.7 ± 5.5	74.9 ± 1.9	69.4 ± 4.6	−34.96

* Normalized with respect to the peptide emission fluorescence at 304 nm in DMSO at 15 °C. ** Experiments were performed at 25 °C with 100 measurement iterations per sample.

**Table 2:** Detection of SARS-CoV-2 on nasopharyngeal swabs by RT-qPCR and biotin-streptavidin-enzyme-linked assay.

	RT-qPCR Detection			bs-ELA
NpS Sample	Ct ORF1ab	Ct β-Actin	Result	Result
P1	7.3	20.2	Positive	Positive
P2	10.3	20.6	Positive	Positive
P3	14.2	22.8	Positive	Positive
P4	22.6	21.2	Positive	Positive
P5	34.3	28.5	Positive	not consistent
N1	no ct	19.4	Negative	Negative
N2	no ct	20.8	Negative	Negative
N3	no ct	21.7	Negative	Negative
N4	no ct	19.8	Negative	Negative
N5	no ct	22.3	Negative	Negative
C+	31.2	27.7		
NTC	no ct	no ct		

## Data Availability

Not applicable.

## Supplementary Materials

The following supporting information can be downloaded at: https://www.mdpi.com/article/10.3390/bios13040473/s1, Figure S1: ACE2 peptide’s tyrosine fluorescence emissions spectra under different conditions. (A) Peptide Tyr fluorescence emissions when exposed to phosphate buffer saline (PBS; pH 7), PBS 10× (pH 7), bicarbonate (BB; pH 8), acetate (pH 5), Tris (pH 8), Hepes (pH 7), and DMSO at 10 % *v*/*v*. (B) Peptide Tyr fluorescence emission when exposed to PBS (pH 7) at three different temperatures. Similar behavior was observed for the other conditions. Samples were excited at 280 nm.
