# Supplementary material for: Stability of ACE2 Peptide Mimetics and Their Implications on the Application for SARS-CoV2 Detection
Source: Biosensors (Basel). 2023 Apr 13;13(4):473. doi: 10.3390/bios13040473 (PMC10136198; doi:10.3390/bios13040473)
Supplement: Supplementary file 1 [file biosensors-13-00473-s001.zip › biosensors-2291832-supplementary.pdf]

biosensors

Supporting Information

Stability of ACE2 Peptide Mimetics and Their Implications on the Application for SARS-CoV2 Detection

Paula A. Santana [1], Claudio A. Álvarez [2,3,*], Santiago Valenzuela [1], Alberto Manchego [4], Fanny Guzmán [5], Cristian Tirapegui [1] and Manuel Ahumada [6,7,*]

[1] Instituto de Ciencias Químicas Aplicadas, Facultad de Ingeniería, Universidad Autónoma de Chile, el Llano Subercaseaux 2801, San Miguel, Santiago 8910272, Chile
[2] Laboratorio de Fisiología y Genética Marina, Centro de Estudios Avanzados en Zonas Áridas, Larrondo 1281, Coquimbo 1780000, Chile
[3] Facultad de Ciencias del Mar, Universidad Católica del Norte, Larrondo 1281, Coquimbo 1780000, Chile
[4] Laboratorio de Microbiología, Facultad de Medicina Veterinaria y Parasitología Veterinaria, Universidad Nacional Mayor de San Marcos, Lima 3673, Peru
[5] Núcleo Biotecnología Curauma, Pontificia Universidad Católica de Valparaíso, Valparaíso 2373223, Chile
[6] Escuela de Biotecnología, Facultad de Ciencias, Ingeniería y Tecnología, Universidad Mayor, Camino La Piramide 5750, Huechuraba, Santiago 8910272, Chile
[7] Centro de Nanotecnología Aplicada, Facultad de Ciencias, Ingeniería y Tecnología, Universidad Mayor, Camino La Piramide 5750, Huechuraba, Santiago 8910272, Chile
* Correspondence: claudio.alvarez@ceaza.cl (C.A.Á.); manuel.ahumada@umayor.cl (M.A.)

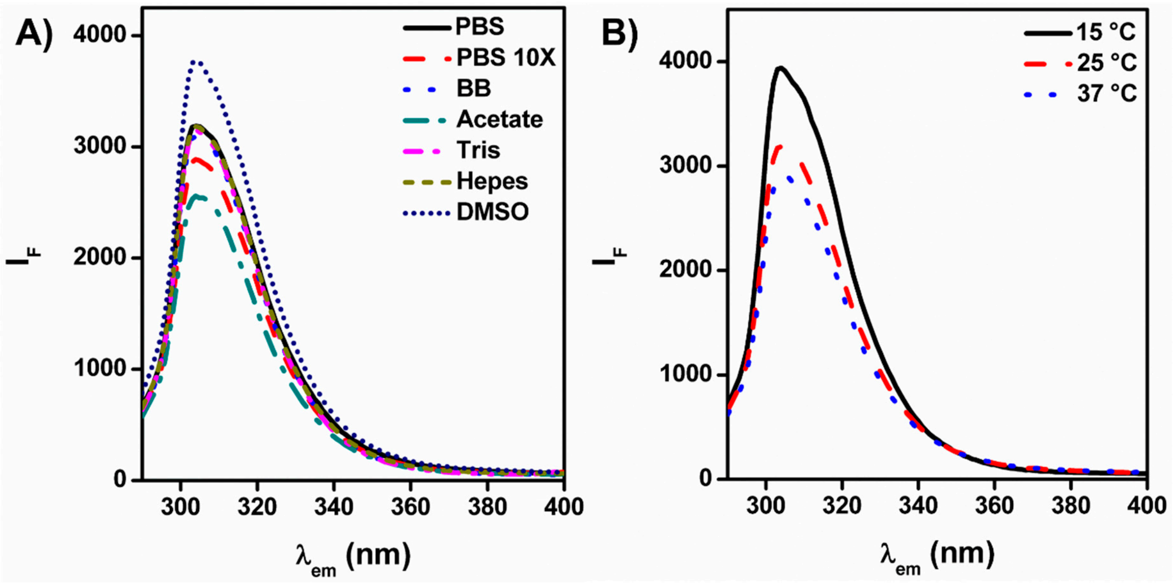

Figure S1. ACE2 peptide´s tyrosine fluorescence emissions spectra under different conditions. (**A**) Peptide Tyr fluorescence emissions when exposed to phosphate buffer saline (PBS; pH 7), PBS 10x (pH 7), bicarbonate (BB; pH 8), acetate (pH 5), Tris-HCl (pH 8), Hepes (pH 7), and DMSO at 10 % v/v. (**B**) Peptide Tyr fluorescence emission when exposed to PBS (pH 7) at three different temperatures. Similar behavior was observed for the other conditions. Samples were excited at 280 nm.
